# Neuroprotective effect of engineered *Clostridium* butyricum‐pMTL007‐GLP‐1 on Parkinson's disease mice models via promoting mitophagy

**DOI:** 10.1002/btm2.10505

**Published:** 2023-03-17

**Authors:** Yun Wang, Wen‐jie Chen, Yi‐yang Han, Xuan Xu, Ai‐xia Yang, Jing Wei, Dao‐jun Hong, Xin Fang, Ting‐tao Chen

**Affiliations:** ^1^ Department of Neurology The First Affiliated Hospital of Nanchang University Nanchang Jiangxi Province P. R. China 330006; ^2^ Institute of Translational Medicine Nanchang University Nanchang Jiangxi Province P. R. China 330031

**Keywords:** *Clostridium butyricum*, genetically engineered strain, GLP‐1, gut microbiota, mitophagy, Parkinson's disease

## Abstract

Parkinson's disease (PD) is a common neurodegenerative disease with limited treatment and no cure, hence, broadening PD drug spectrum is of great significance. At present, engineered microorganisms are attracting increasing attention. In this study, we constructed an engineered strain of *Clostridium butyricum*‐GLP‐1, a *C. butyricum* (a probiotic) that consistently expresses glucagon‐like peptide‐1 (GLP‐1, a peptide‐based hormone with neurological advantage) in anticipation of its use in PD treatment. We further investigated the neuroprotective mechanism of *C. butyricum*‐GLP‐1 on PD mice models induced by 1‐methyl‐4‐phenyl‐1,2,3,6‐tetrahydropyridine. The results indicated that *C. butyricum*‐GLP‐1 could improve motor dysfunction and ameliorate neuropathological changes by increasing TH expression and reducing the expression of α‐syn. Moreover, we confirmed that *C. butyricum*‐GLP‐1 improved microbiome imbalance of PD mice by decreasing the relative abundance of *Bifidobacterium* at the genus level, improved gut integrity, and upregulated the levels of GPR41/43. Surprisingly, we found it could exert its neuroprotective effects via promoting PINK1/Parkin mediated mitophagy and attenuating oxidative stress. Together, our work showed that *C. butyricum*‐GLP‐1 improves PD by promoting mitophagy, which provides an alternative therapeutic modality for PD.

## 1 INTRODUCTION

Parkinson's disease (PD) ranks the second most common neurodegenerative disease with rapid ongoing prevalence undergoing.^1^ PD patients suffer from progressive motor dysfunction and cognitive impairment, bringing great misery to life quality and their families. Unfortunately, as a polyfactorial disease, the pathogenesis of PD is poorly understood with very limited therapeutics.^2^ Current first‐line drugs include dopamine precursors, dopamine receptor agonists, and amantadine that are mainly symptomatic treatment that aims to reduce PD‐associated dopamine reduction, which may be impossible to halt or reverse disease progression.^3^ Consequently, finding drugs that are able to cure PD aggravation as well as broaden PD therapeutic spectrum is of great importance.

Growing research supports that mitochondrial dysfunction is genetically and pathologically linked to PD.^4^ It was found that mitochondria in central nervous system of PD underwent morphological changes and functional loss in post‐mortem brains from sporadic PD patients.^5^ Moreover, mitochondrial damage in PD can lead to further apoptosis of dopaminergic neurons.^6^ The regulator of mitochondrial fate is mitophagy, which clears the damaged mitochondria via lysosome‐mediated degradation. Mitochondrial damage and defective mitophagy have been found in in and ex vivo PD models.^7^ Besides, several PD‐associated proteins have been demonstrated to play a role in the regulation of mitochondrial autophagy, such as PTEN‐induced kinase 1 (PINK1) and Parkin RBR E3 ubiquitin‐protein ligase (Parkin). Prior work has found that PINK1/Parkin pathway is a classic mitophagy pathway, with PINK1 being able to accumulate on damaged mitochondria, thereby inducing mitochondrial autophagy.^8^ Mutations in PINK1 and Parkin prevent the elimination of damaged mitochondria, thereby triggering the development of PD.^9^ Therefore, mitophagy enhancement may be a potential resolution for PD treatment.

Increasing studies confirmed that gut microbiota is associated with PD,^10^, ^11^ and study found an existence of intestinal dysbiosis in PD patients, whose specific manifestation was the increased abundance of Enterobacteriaceae at phylum level and *Lactobacillus* at genus level, as well as the reduced abundance of *Prevotella* at genus level.^12^ In addition, fecal transplantation from PD patients to germ‐free mice was reported to result in motor deficit in mice.^13^ *Clostridium butyricum* (*C. butyricum*) is a probiotic strain that may survive in the digestive tract and has a variety of therapeutic benefits.^14^ Previous work has shown that the *C. butyricum* treatment inhibited the neuroinflammation of Alzheimer's disease by modulating intestinal microbiota,^15^ and performed a positive effect on vascular dementia^16^ and ischemia/reperfusion injury.^17^ Besides, another work has found that *C. butyricum* ameliorated motor deficits by gut microbiota‐glucagon‐like peptide‐1 (GLP‐1) pathway in PD mice,^18^ suggesting *C. butyricum* might alleviate the symptoms of PD.

GLP‐1 is an incretin hormone secreted by ileal endocrine cells.^18^ Previous work indicated that GLP‐1 can provide protective impacts on the central nervous system via modulating neuronal cell production and neuroapoptosis.^19^, ^20^ However, dipeptidyl peptidase‐IV (DPP‐IV, CD26) can quickly hydrolyze functional GLP‐1, resulting in half‐life of GLP‐1 at less than 2 min.^21^ GLP‐1 receptor (GLP‐1R) agonists have received great effectiveness in clinical trials.^22^, ^23^ Liraglutide, with a 97% homology and a 13‐h half‐life, is the first GLP‐1R agonist derived from natural GLP‐1.^24^ Notably, liraglutide crosses the blood–brain barrier and enters the central nervous system. A randomized, double‐blind, placebo‐controlled trial showed that liraglutide can improve motor function and daily activities in patients with PD.^25^ In addition, several animal experiments have also verified that liraglutide has a neuroprotective effect on PD.^26^, ^27^, ^28^ Whereas, commercial GLP‐1 analogous requires continuous injection, leading to bad compliance. Consequently, this study intends to integrate GLP‐1 coding gene sequence into the genome of *C. butyricum* to construct an engineered strain that can combine the neuroprotective efficacy of GLP‐1 with the probiotic properties of *C. butyricum*.

In this trial, the neuroprotective effect of *Clostridium butyricum*‐pMTL007‐GLP‐1 (*C. butyricum*‐GLP‐1) was assessed in PD mice models and its underlying mechanism was explored via behavioral tests, immunohistochemistry, immunofluorescence, ultrastructural morphology, Western blotting, and 16S rDNA high throughput sequencing. We hope the results of our study will shed fresh light on neurodegeneration and assist developing PD‐protecting medications.

## 2 MATERIALS AND METHODS

### 2.1 GLP‐1 expression and growth characteristics of *C.* *butyricum*‐GLP‐1 in vitro

The bacterial strain *C. butyricum* (NCU‐02, CGMCC, no. 25504) and the genetically engineered strain *C. butyricum*‐GLP‐1 were from lab stock. Briefly, the engineered strain contains hGLP‐1 gene at 5′ HindIII to 3′ BsrGI site in pMTL007 (Professor Hongjun Dong, Tianjin Industrial Biology, Chinese Academy of Sciences) plasmid specialized for *C. butyricum*. *C. butyricum*‐GLP‐1 was grown in Tryptic Soy Broth (TSB, Solarbio) with thiamphenicol at 37°C under anaerobic conditions. Then, GLP‐1 concentration in supernatant was detected by human GLP‐1 enzyme‐linked immunosorbent assay (ELISA) kit (ELabscience, E‐EL‐H6025) following protocol offered by the manufacturer. The growth curve assay was performed by detecting bacterial growth with light absorption at 600 nm at 2‐h intervals over 24 h. For the acid tolerance test, equal amounts of bacteria were centrifuged and incubated with PBS at different pH (including 2, 3, 4, 5, 7) for 4 h and viable bacteria were determined by spot counting. For the bile salt test, the procedure was the same as for the acid test except that the pH was changed to 0.0%–0.5% bile salt.^29^

### 2.2 Animals and experimental design

Forty C57BL/6 eight‐week‐old male mice (22–28 g) were acquired from Hunan SJA Laboratory Animal. The mice were raised in an SPF animal house with 12/12 light cycle, humidity of 50%–55%, and at 22–24°C. Mice have unrestricted access to water and food. To eliminate mistakes due to experimental time, all trials were conducted between 9:00 and 12:00 a.m. Figure 2a depicts the animal treatment schedule. First, after a week of adapting housing, 8 mice were randomly selected from 40 mice when control group (C group) was given saline gelatin for 14 days. The remaining mice were given 20 mg/kg 1‐methyl‐4‐phenyl‐1,2,3,6‐tetrahydropyridine (MPTP; Sigma‐Aldrich, M0896) intraperitoneally for 7 days, and then divided into four groups of eight mice each as follows: model group (M group) received saline gelatin treatment for 7 days; liraglutide group (L group) received an intraperitoneal injection of 0.4 mg/kg liraglutide (J20160037) for 7 days; *C. butyricum* group (CB group) received 10^8^ CFU/mL *C. butyricum* gavage (resuspended in saline containing 0.01% gelatin) for 7 days; *C. butyricum*‐GLP‐1 (CBG group) received 10^8^ CFU/mL *C. butyricum*‐GLP‐1 gavage (resuspended in saline containing 0.01% gelatin) for 7 days. All procedures were conducted in accordance with the guidelines and regulations of the National Institutes of Health and were approved by the Laboratory Animal Ethics Committee of Nanchang Royo Biotechnology Co., Ltd, Nanchang, China (Approval Number: RYE2021060401).

### 2.3 Behavioral assessment

The pole test, open‐field test, and hanging wire test were included for behavioral assessment. In the pole test, a cotton‐wrapped stick was prepared and the mice were placed at the top, and corresponding time taken to descend from the top to the bottom was recorded. In the open‐field test, the total distance traveled and the distance traveled in the central area were recorded over 10‐min period.^30^ In the hanging wire test, the two front paws of the mouse were placed in the middle of the rope and the number of limbs the mouse used to grab the thin rope was recorded: the number of paws able to grab the rope was recorded as a score, if the mouse dropped off, then mark 0. Each test was taken with 15 min interval to rest, then the average number of three tests per mouse would be recorded for later analysis.^31^

### 2.4 Sample collection

After the behavioral tests were finished, mice were euthanized using isoflurane gas as anesthesia.^32^ Then, the collected blood was clotted at room temperature for 2 h and centrifuged. The serum was stored at −80°C. Fecal samples were stored at −80°C. Brain and colonic tissues were preserved at −80°C or fixed with 4% paraformaldehyde for histopathological assays.

### 2.5 Immunohistochemistry (IHC) and immunofluorescence (IF)

The fixed brain and colonic tissue were paraffin‐embedded and cut into 5 μm thick sections. Next, the sections were deparaffinized and incubated with 3% H_2_O_2_ and 5% goat serum subsequently at room temperature. Tissues were then incubated overnight at 4°C with primary antibodies (Table SS1). Sections were then incubated with secondary antibody to label binding sites.

### 2.6 Transmission electron microscopy (TEM)

The substantia nigra (SN) was fixed in 2.5% glutaraldehyde at 4°C for 4 h. Tissue pellets were then treated in 1% phosphate buffered OsO_4_ solution (pH = 7.4) and serially dried in acetone and resin‐embedded. TEM was used to examine ultrathin slices (60–80 nm) stained with uranyl and lead salt (HITACHI; HT7800).

### 2.7 Gut microbiota analysis

The gut microbiota was analyzed by 16S rRNA high throughput sequencing as previously described.^30^ Briefly, bacterial genomic DNA was first extracted and amplified (Personalbio). Then, after paired‐end sequencing, the data were analyzed by QIIME2 (version 2019.4). The relative abundance of operational taxonomic units (OTUs) in at least six samples for assays were examined, including species composition, α‐diversity, and β diversity (raw data available on NCBI, PRJNA893872).

### 2.8 Western blotting analysis

The Western blotting experiment was performed as described previously. In short, total protein in brain and colonic tissue was extracted and separated by SDS‐PAGE. After being transferred onto polyvinylidene fluoride membrane and blocked, the membrane was incubated with primary antibodies (Table SS1) at 4°C overnight. Then, the membrane was further incubated with secondary antibody at room temperature for 90 min after washed three times with TBST. The protein level was examined by imaging system with chemiluminescence detection.

### 2.9 Measurement of oxidative stress (OS) associated biomarkers and GLP‐1

The activity of glutathione peroxidase (GSH‐Px), malondialdehyde (MDA) content, and superoxide dismutase (SOD) in the SN and serum, and the GLP‐1 level in serum was evaluated by corresponding assay kit (Nanjing Jiancheng Bioengineering Institute, A005‐1‐2, A005‐1‐2, A001‐3‐2, and IBL, 27788 respectively) following protocol offered by the manufacturer.

### 2.10 Data analysis

Data analysis was performed with Prism version 7.0 (GraphPad Software, San Diego, CA, USA). Experimental data were statistically examined by one‐way analysis of variance (ANOVA) followed by Tukey's test for multiple comparisons. Microbiota data were analyzed by Kruskal–Wallis rank sum test and Dunn's test as post hoc tests. Results were presented in the form of mean ± standard deviation (SD), and *p* < 0.05 was regarded to be statistically significant (*p < 0.05, **p < 0.01).

## 3 RESULTS

### 3.1 Microbial characteristics of *C.* *butyricum*‐GLP‐1 in vitro

**FIGURE 1:**
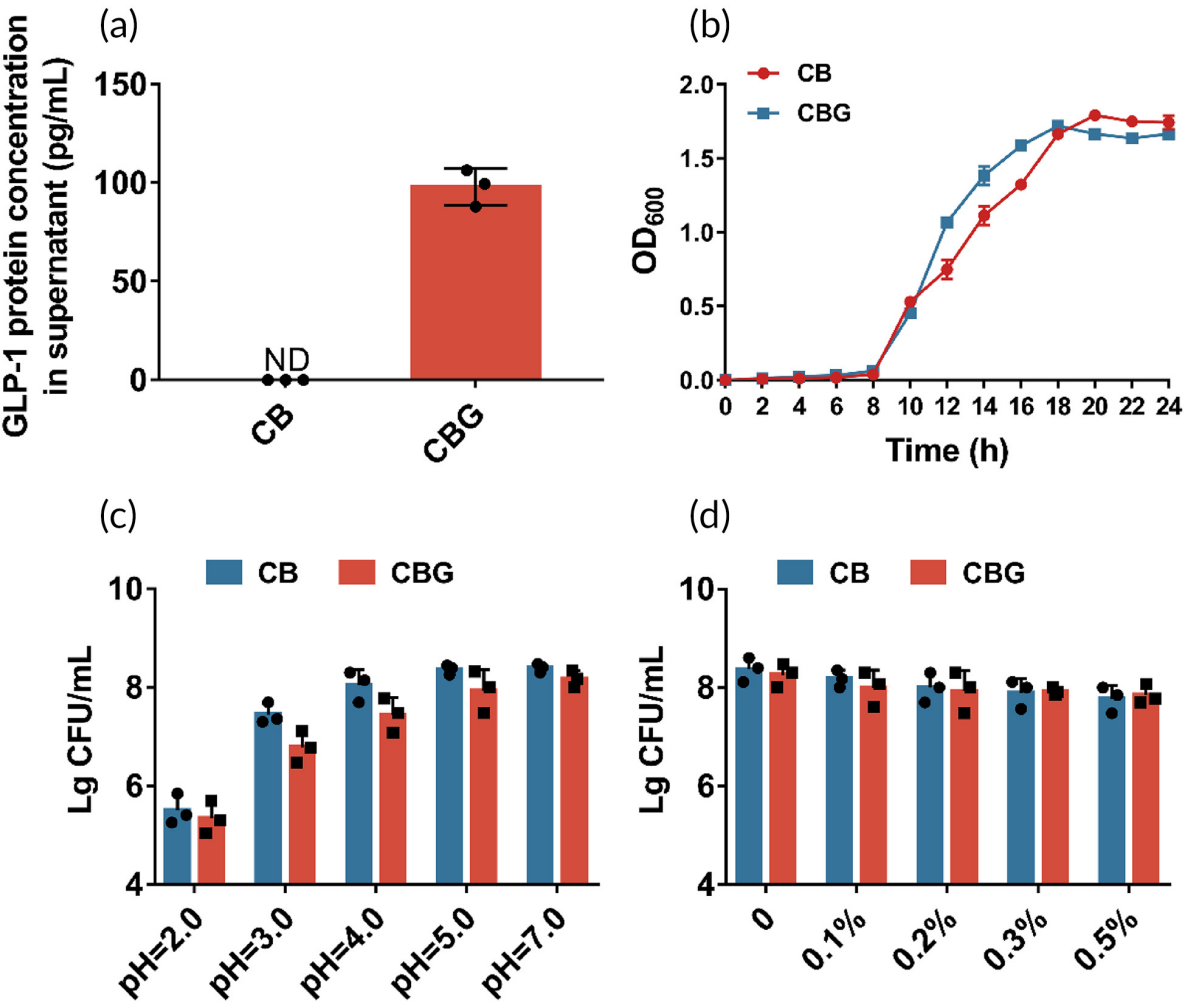
Evaluation of probiotic characteristics of *C. butyricum*‐GLP‐1 in vitro. (a) ELISA analysis of GLP‐1 production capability of *C. butyricum*‐GLP‐1 (300 mL system). (b) Growth curves of *C. butyricum* and *C. butyricum*‐GLP‐1. (c) Acid tolerance of *C. butyricum* and *C. butyricum*‐GLP‐1. (d) Cholate tolerance of *C. butyricum* and *C. butyricum*‐GLP‐1. CB, *Clostridium butyricum*; CBG, *C. butyricum*‐GLP‐1; ELISA, enzyme‐linked immunosorbent assay; GLP‐1, glucagon‐like peptide 1.

First, we evaluated the GLP‐1 production capability of *C. butyricum*‐GLP‐1, and found this strain can yield 97.97 ± 9.295 pg/mL GLP‐1 in supernatant by ELISA (Figure 1a). Compared with the wild strain *C. butyricum*, no significant difference in growth characteristics between *C. butyricum* and *C. butyricum*‐GLP‐1 was observed, although *C. butyricum*‐GLP‐1 reached its peak growth 2 h earlier than *C. butyricum* (Figure 1b). Finally, we investigated the probiotics’ characteristics of *C. butyricum*‐GLP‐1, and found that both *C. butyricum*‐GLP‐1 and *C. butyricum* have a sound tolerance to acid and bile salts, which ensure their survival in host stomach and intestinal tract (Figure 1c,d).

### 3.2 *C. butyricum*‐GLP‐1 improved motor dysfunction in the PD mice

**FIGURE 2:**
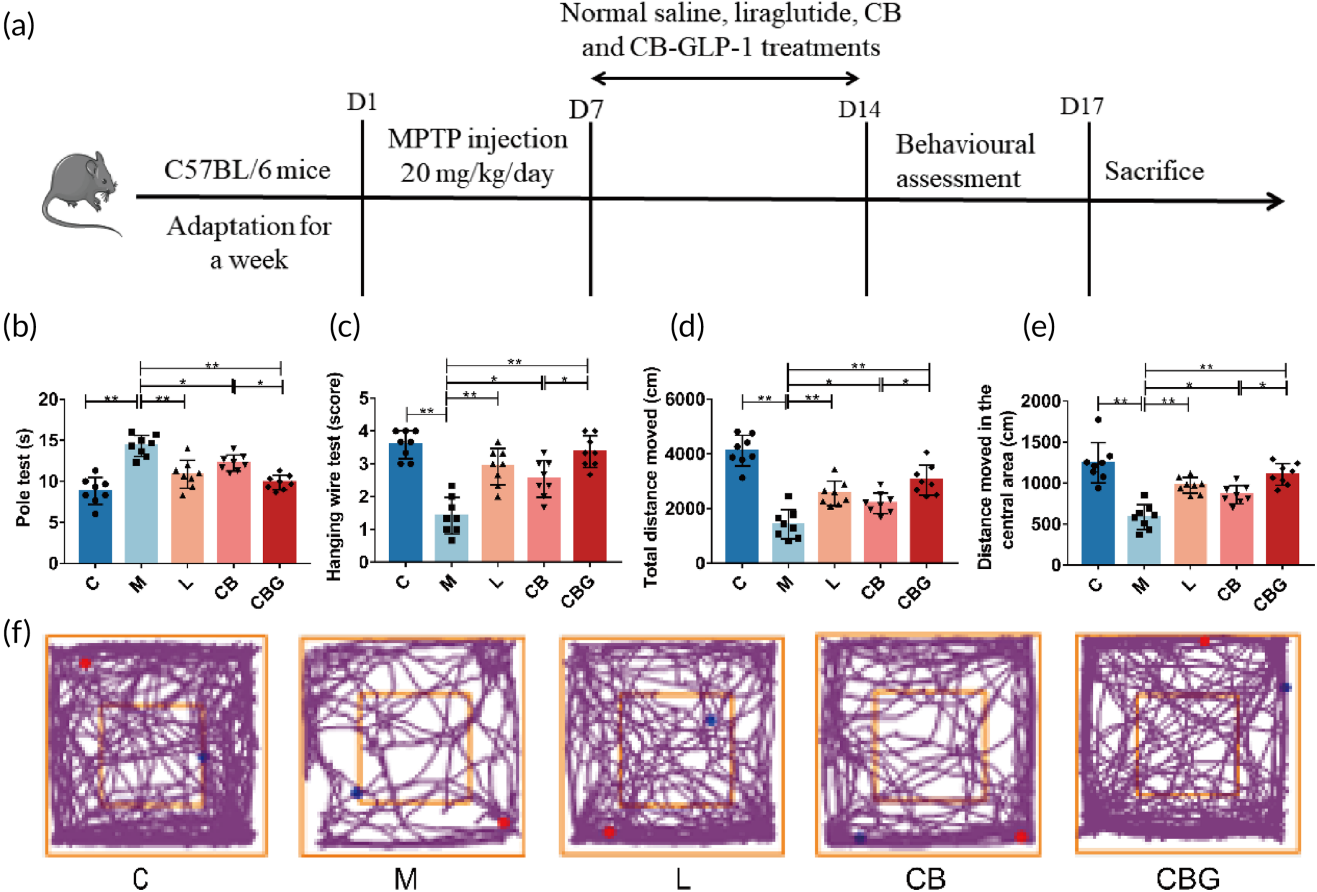
*C. butyricum*‐GLP‐1 ameliorated the motor impairment caused by MPTP in PD mice. (a) Schematic workflow of the whole experiment. (b) Pole test, *n* = 8. (c) Hanging wire test, *n* = 8. (d) The total distance moved in open‐field test, *n* = 8. (e) The distance in the central area moved in open‐field test, *n* = 8. (f) Representative traces of the open field test. (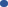): starting position; (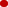), ending position. C group, control group; M group, MPTP group; L group, MPTP + liraglutide group; CB group, MPTP + CB group; CBG group, MPTP + CB‐GLP‐1 group. CB, *Clostridium butyricum*; CBG, *C. butyricum*‐GLP‐1; GLP‐1, glucagon‐like peptide 1; MPTP, 1‐methyl‐4‐phenyl‐1,2,3,6‐tetra‐hydropyridine; PD, Parkinson's disease.

Next, MPTP‐induced PD mice models were constructed to further evaluate the effect of *C. butyricum*‐GLP‐1 on the motor ability by behavioral tests. The pole test result indicated that the decreased locomotor ability observed in M group was greatly improved by *C. butyricum*‐GLP‐1 (CBG vs. M = 9.875 ± 0.8898 s vs. 14.33 ± 1.272 s, *p* < 0.01) or liraglutide treatment (L vs. M = 10.87 ± 1.708 s vs. 14.33 ± 1.272 s, *p* < 0.01). Simultaneously, there was no statistical difference between *C. butyricum*‐GLP‐1 and liraglutide (CBG vs. L = 9.875 ± 0.8898 s vs. 10.87 ± 1.708 s, *p >* 0.05). Interestingly, the result found that the *C. butyricum* alone had shown improved behavior (*C. butyricum* vs. M = 12.25 ± 0.9718 s vs. 14.33 ± 1.272 s, *p* < 0.05), but this difference was slight (Figure 2b). The results of hanging wire test revealed that *C. butyricum*‐GLP‐1 displayed the same tendency as that of the pole test (Figure 2c), suggesting that *C. butyricum*‐GLP‐1 could improve muscle strength and balance in PD mice. Finally, in the open field test, mice in the M group showed lower exploratory ability than the C group. The same tendency was observed in *C. butyricum*‐GLP‐1 group (Figure 2f). Specifically, the total distance traveled (CBG vs. M = 3041 ± 549.2 cm vs. 1425 ± 537.5 cm, *p <* 0.01) and the distance traveled in the center area (CBG vs. M = 1106 ± 131.1 cm vs. 584.1 ± 151.2 cm, *p <* 0.01) were highly increased (Figure 2d,e). Together, these results demonstrated that *C. butyricum*‐GLP‐1 could improve motor dysfunction in PD mice.

### 3.3 *C. butyricum*‐GLP‐1 alleviated neuropathological changes in PD mice

**FIGURE 3:**
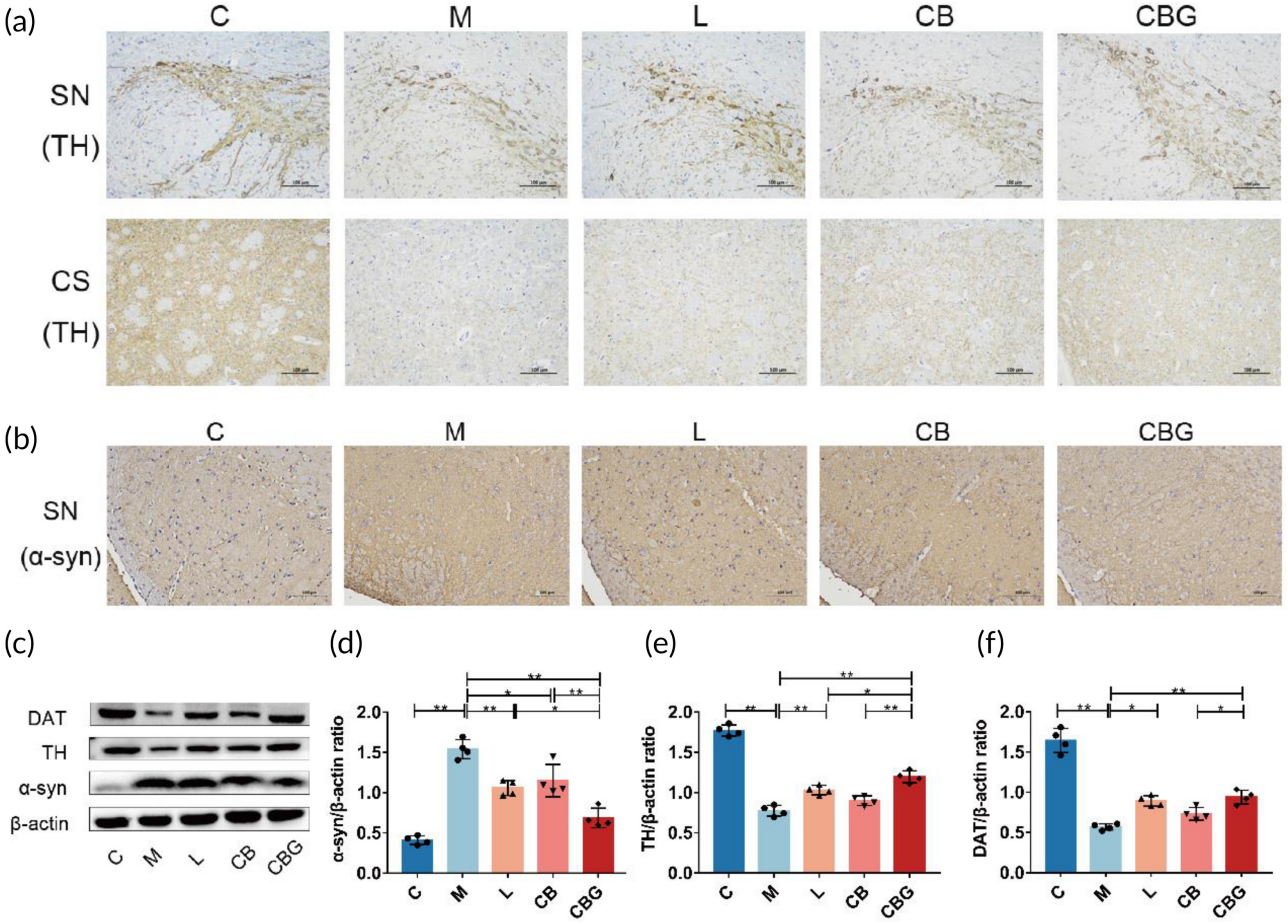
*C. butyricum*‐GLP‐1 ameliorated neuropathological alterations produced by MPTP in PD mice. (a) Representative IHC analysis of TH; the upper image in the SN and the lower image in the CS (scale bar = 100 μm). (b) Representative IHC analysis of α‐syn (scale bar = 100 μm). (c) Western blotting of DAT, TH, α‐syn, and β‐actin expression in the SN. (d–f) Quantitative analysis of western blots for α‐syn (d), TH (e), and DAT (f), *n* = 4. α‐syn, alpha‐synuclein; CS, corpus striatum; DAT, dopamine transporter; GLP‐1, glucagon‐like peptide 1; IHC, immunohistochemistry; MPTP, 1‐methyl‐4‐phenyl‐1,2,3,6‐tetra‐hydropyridine; PD, Parkinson's disease; SN, substantia nigra; TH, tyrosine hydroxylase.

To investigate the pathological alteration of *C. butyricum*‐GLP‐1 in PD mice, the expression of TH was evaluated by IHC and Western blotting. In the M group, there was severe TH‐positive cell decrement in the SN and corpus striatum (CS), which was improved by treating with liraglutide, *C. butyricum*, and *C. butyricum*‐GLP‐1 (Figure 3a). In addition, α‐syn, a pathological marker of PD, was increased in the M group, whereas, *C. butyricum*‐GLP‐1 substantially diminished α‐syn (Figure 3b). The Western blotting further supported the morphological finding (Figure 3c–f), and the expression of α‐syn was downregulated while TH was particularly upregulated in CBG group. Moreover, DAT, an important protein for PD, showed the same trend as TH. Thus, these results revealed that the *C. butyricum*‐GLP‐1 might ameliorate neuropathology by downregulating nigral α‐syn level, and increasing TH and DAT level in PD mice.

### 3.4 *C. butyricum*‐GLP‐1 increased the level of GLP‐1 and GLP‐1R in PD mice

**FIGURE 4:**
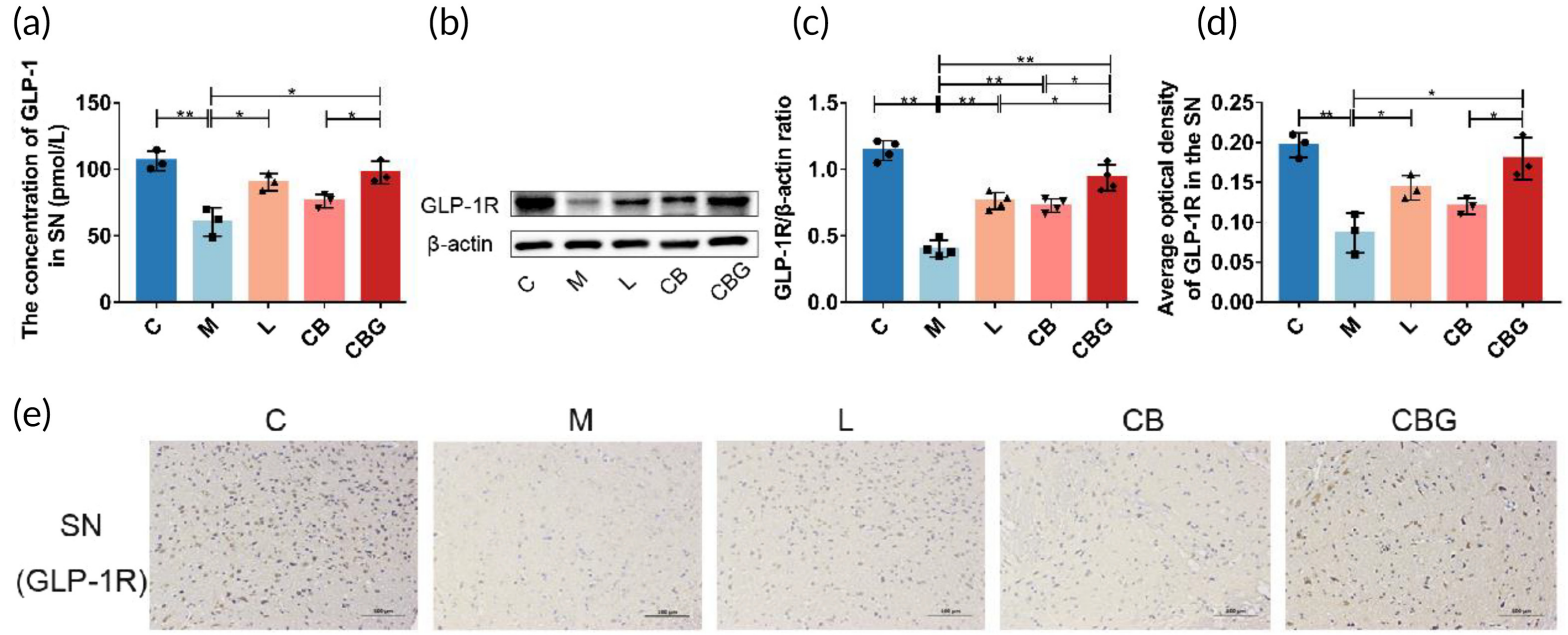
*C. butyricum*‐GLP‐1 might enhance GLP‐1 and GLP‐1R levels in PD mice. (a) ELISA analysis of the concentration of GLP‐1 in the SN, *n* = 3. (b) Western blotting of GLP‐1R and β‐actin expression in the SN. (c) Quantitative analysis of Western blotting of GLP‐1R. (d) Quantification analysis of immunohistochemistry of the content of GLP‐1R. (e) Immunohistochemistry analysis of GLP‐1R (scale bar = 100 μm), *n* = 4. ELISA, enzyme‐linked immunosorbent assay; GLP‐1, glucagon‐like peptide 1; GLP‐1R, GLP‐1 receptor.

To verify whether the improved behavior is associated with exogenous GLP‐1, GLP‐1 concentration was evaluated in the SN using ELISA assay (Figure 4a). The results revealed that MPTP markedly decreased GLP‐1 concentration in contrast to C group (60.45 ± 10.67 pmol/L vs. 106.5 ± 7.379 pmol/L, *p* < 0.01), while liraglutide (90.36 ± 6.366  pmol/L vs. 60.45 ± 10.67 pmol/L, *p* < 0.05) and *C. butyricum*‐GLP‐1 (97.57 ± 8.461 pmol/L vs. 60.45 ± 10.67 pmol/L, *p* < 0.05) treatment increased GLP‐1 concentration. Then, Western blotting and IHC were respectively performed to detect the expression and distribution of GLP‐1R. Results revealed that MPTP treatment substantially suppressed the expression of GLP‐1R. However, the liraglutide and *C. butyricum*‐GLP‐1 treatment vastly upregulated the expression of GLP‐1R (Figure 4b–e). Together, these results suggested that *C. butyricum*‐GLP‐1 increased the concentration of GLP‐1 and GLP‐1R in PD mice.

### 3.5 *C. butyricum*‐GLP‐1 promoted PINK1/Parkin‐mediated mitophagy pathway in PD mice

**FIGURE 5:**
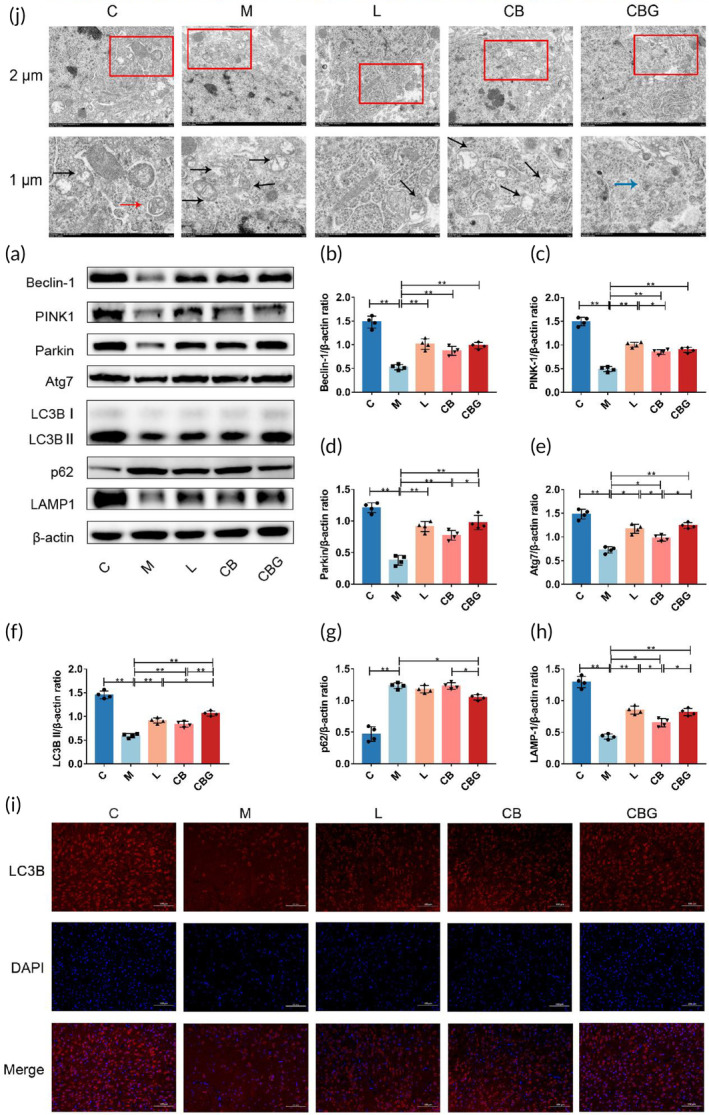
*C. butyricum*‐GLP‐1 may ameliorate PD through the PINK1/Parkin mitophagy pathway. (a) Western blotting of LAMP‐1, Atg7, PINK1, Beclin‐1, LC3B II, p62, Parkin, and β‐actin expression in the SN. Quantitative analysis of western blots for Beclin‐1 (b), PINK1 (c), Parkin (d), Atg7 (e), LC3B II (f), p62 (g), and LAMP‐1 (h), *n* = 4. Immunofluorescence analysis of LC3B (i). The image above in the SN (scale bar = 100 μm). Representative TEM image of mitophagy in the SN of mice (j). The image above: scale bar = 2 μm. The image below: scale bar = 1 μm (the red arrow pointed to autophagosomes, the black arrow pointed to damaged mitochondria, and the blue arrow pointed to autophagic lysosomes). Atg7, autophagy related 7; GLP‐1, glucagon‐like peptide 1; LAMP‐1, lysosomal‐associated membrane protein 1; Parkin, Parkin RBR E3 ubiquitin‐protein ligase; PD, Parkinson's disease; PINK1, PTEN‐induced kinase 1; SN, substantia nigra; TEM, transmission electron microscopy.

Defective mitochondrial phagocytosis is linked to the pathogenesis of PD,^33^ and several studies have found that promoting PINK1/Parkin‐mediated mitophagy could protect dopaminergic (DAergic) neurons in PD.^7^, ^34^ To further determine whether *C. butyricum*‐GLP‐1 exerts neuroprotective effects via modulating the PINK1/Parkin pathway, the immunoblotting of the expressions of key proteins involved were performed (Figure 5a–h). The results showed that *C. butyricum*‐GLP‐1 alleviated the down‐expression of Beclin‐1, PINK1, Parkin, Atg7, LC3B II, and LAMP‐1 and increased the expression of p62 caused by MPTP. In addition, IF was used to prove our findings by detecting the positive neurons for LC3B, an autophagosome membrane marker protein.^35^ As shown in Figure 5i, *C. butyricum*‐GLP‐1 prevented the reduction of LC3B expression. In order to further verify our findings, the ultrastructural pathology of the mitochondria was evaluated using the TEM (Figure 5j). The result found that there was more mitochondrial destruction, fewer autophagosomes and autolysosome in the M group, which is improved by *C. butyricum*‐GLP‐1 treatment. Therefore, these results suggested that *C. butyricum*‐GLP‐1 may promote the PINK1/Parkin mitophagy pathway to clear abnormal mitochondria.

### 3.6 *C. butyricum*‐GLP‐1 alleviated OS in PD mice

**FIGURE 6:**
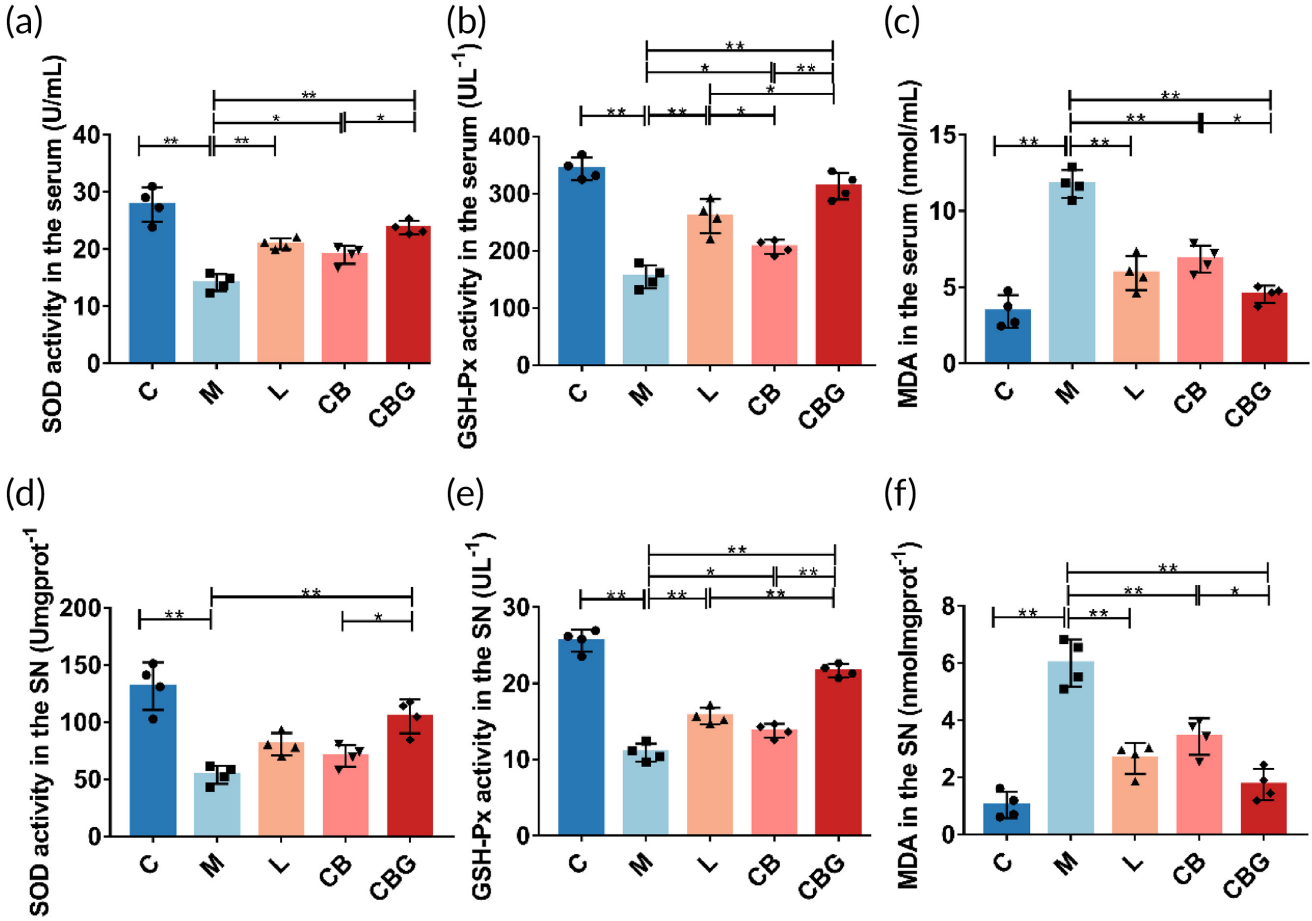
*C. butyricum*‐GLP‐1 alleviated oxidative stress in PD mice. The activity of SOD (a), GSH‐Px (b), and MDA (c). The level of oxidative stress in the SN, the activity of SOD (d), GSH‐Px (e), and MDA (f), *n* = 4. GLP‐1, glucagon‐like peptide 1; GSH‐Px, glutathione peroxidase; MDA, malondialdehyde; PD, Parkinson's disease; SOD, superoxide dismutase.

Related studies have shown that mitochondrial damage can lead to OS.^36^ To determine the role of *C. butyricum*‐GLP‐1 on OS in PD mice, the activities of GSH‐Px, SOD, and MDA in the serum and SN were measured, respectively. In the serum (Figure 6a–c), results showed that MPTP could markedly decrease the activity of SOD compared with C group (14.18 ± 1.477 U/mL vs. 27.81 ± 3.028 U/mL, *p* < 0.01) and GSH‐Px (154.5 ± 20.24 U/L vs. 343.6 ± 19.39 U/L, *p* < 0.01), and substantially increased the level of MDA (11.77 ± 0.9025 nmol/mL vs. 3.419 ± 1.061 nmol/mL, *p* < 0.01). While liraglutide, *C. butyricum*, and *C. butyricum*‐GLP‐1 increased the level of SOD (20.92 ± 0.9503 U/mL, *p* < 0.01; 19.01 ± 1.565 U/mL, *p* < 0.05; 23.78 ± 1.184 U/mL, *p* < 0.01, respectively) and GSH‐Px (260.6 ± 29.94 U/L, *p* < 0.01; 206.8 ± 12.59 U/L, *p* < 0.05; 313.2 ± 23.18 U/L, *p* < 0.01, respectively). At the same time, oxidation product MDA (5.935 ± 1.12 nmol/mL, *p* < 0.01; 6.839 ± 0.9001 nmol/mL, *p* < 0.01; 4.548 ± 0.5612 nmol/mL, *p* < 0.01, respectively) was decreased greatly in the CBG group. The tendency was similar in the SN (Figure 6d–f). Together, these results demonstrated that *C. butyricum*‐GLP‐1 alleviated MPTP‐induced OS.

### 3.7 *C. butyricum*‐GLP‐1 improved intestinal microbiome in PD mice

**FIGURE 7:**
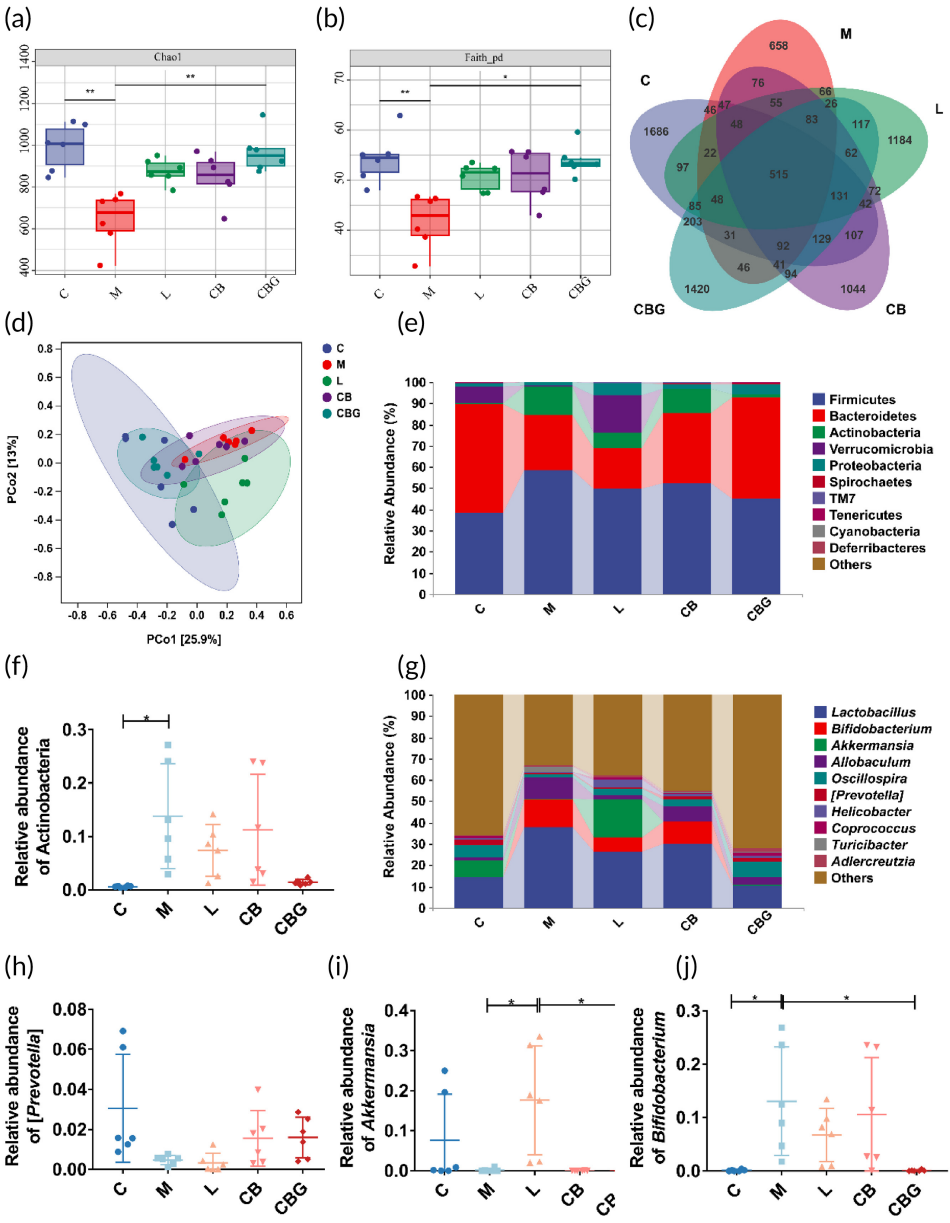
*C. butyricum*‐GLP‐1 recovered the imbalance of the microbiome. (a) The Chao1 index, *n* = 6. (b) The Faith_pd index, *n* = 6. (c) Venn map of OTUs. (d) PCoA of β diversity index. (e) Microbiota composition at the phylum level. (f) The relative abundance of Actinobacteria. (g) Microbiota composition at the genus level. The relative abundance of *Prevotella* (h), *Akkermansia* (i), and *Bifidobacterium* (j), *n* = 6. GLP‐1, glucagon‐like peptide 1; OTUs, operational taxonomic units.

Several studies have confirmed that *C. butyricum* administration improved the intestinal microbiota of PD. Therefore, to inquire into the influence of *C. butyricum*‐GLP‐1 on the intestinal microbiota, the 16S rDNA high throughput sequencing was performed. The results revealed that the α‐diversity included Chao1 index and Faith_pd index were greatly decreased in the M group in contrast to the C group. Nonetheless, *C. butyricum*‐GLP‐1 increased α‐diversity (Figure 7a,b). The Venn analysis found that 515 common OTUs were found in every group, and 1686, 658, 1184, 1044, and 1420 unique OTUs were found in the C, M, L, CB, and CBG groups, respectively (Figure 7c). The result of PCoA analysis revealed that sample points in the M group were away from those in the C group, whereas, samples points in the CBG group were nearer to those in the C group as well as far from those in the M group, which indicated that MPTP extremely changed the β diversity compared to the C group, and the β diversity of the *C. butyricum*‐GLP‐1 treatments was different from that in the M group (Figure 7d). At the phylum level, Firmicutes, Bacteroidetes, Actinobacteria, and Verrucomicrobia were the four most common dominant phyla in these five groups (Figure 7e). Besides, we examined the relative abundance of PD‐related probiotics and pathogens. The MPTP greatly increased the relative abundance of Actinobacteria in the M group relative to the C group (*p* < 0.05), *C. butyricum*‐GLP‐1 treatment decreased this change, but there was no statistical difference (Figure 7f). At the genus level (Figure 7g–j), MPTP diminished the abundance of *Prevotella* and substantially increased the abundance of *Bifidobacterium* relative to the C group. Whereas, *C. butyricum*‐GLP‐1 treatment recovered the decrease of *Bifidobacterium* abundance (*p* < 0.05). Accordingly, these results proved that *C. butyricum*‐GLP‐1 could restore intestinal microbiome in PD mice.

### 3.8 *C. butyricum*‐GLP‐1 restored gut integrity and upregulated GPR41/43 levels in PD mice

**FIGURE 8:**
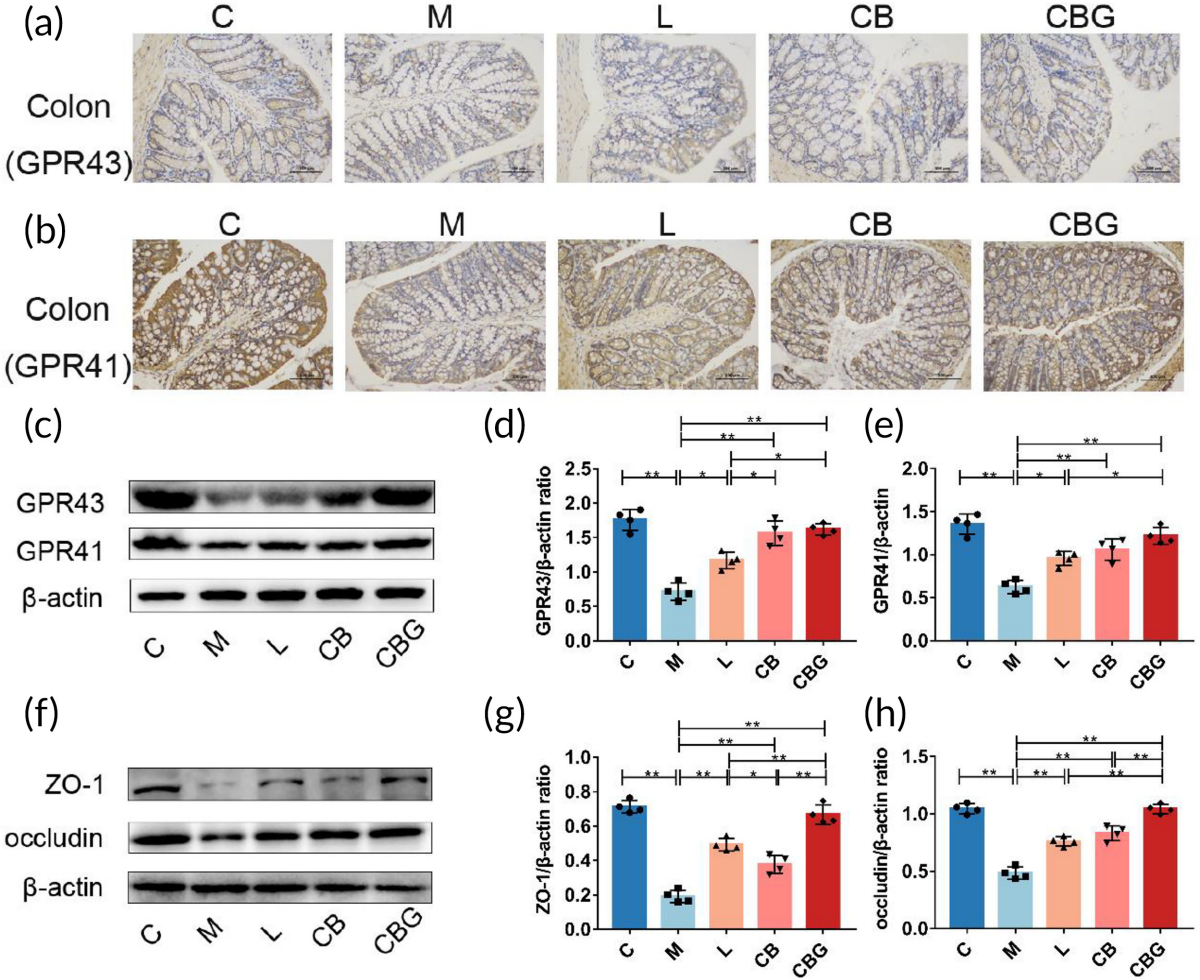
*C. butyricum*‐GLP‐1 recovered the expression of GPR41/43 and intestinal tight junction proteins in PD mice. IHC analysis of GPR43 (a) and GPR41 (b) (scale bar = 100 μm). (c) Western blotting of GPR41, GPR43, and β‐actin expression in the colon. (d and e) Quantitative analysis of Western blotting of GPR41/43, *n* = 4. (f) Western blotting of ZO‐1, occludin, and β‐actin expression in the colon. (g and h) Quantitative analysis of Western blotting of ZO‐1 (g) and occludin (h), *n* = 4. GLP‐1, glucagon‐like peptide 1; GPR43, G‐protein‐coupled receptor 43; GPR41, G protein‐coupled receptor 41; IHC, immunohistochemistry; PD, Parkinson's disease; ZO‐1, Zona occludens 1.

Recent studies have found that *C. butyricum* exerts neuroprotective effects via GPR41/43,^18^ which are the receptors for butyric acid. Hence, to evaluate the expression of GPR41/43, IHC and Western blotting were performed respectively (Figure 8a,b). The results revealed that MPTP substantially reduced GPR41 and GPR43‐positive cells in comparison to the C group, while, the liraglutide, *C. butyricum*, and *C. butyricum*‐GLP‐1 increased GPR41/43 positive cells. The Western blotting further supported the morphological finding (Figure 8c–e). In addition, butyric acid has a strong ability to repair mucosa. To assess the expression of intestinal tight junction proteins (ZO‐1 and occludin) in the colon, Western blotting was performed (Figure 8f–h). The results proved that MPTP greatly inhibited the expression of ZO‐1 and occludin contrast to the C group, however, the liraglutide, *C. butyricum*, and *C. butyricum*‐GLP‐1 recovered these changes, particularly in CBG group (*p* < 0.01). Therefore, these results revealed that *C. butyricum*‐GLP‐1 recovered the levels of GPR41/43 and intestinal tight junction proteins in PD mice.

## 4 DISCUSSION

**FIGURE 9:**
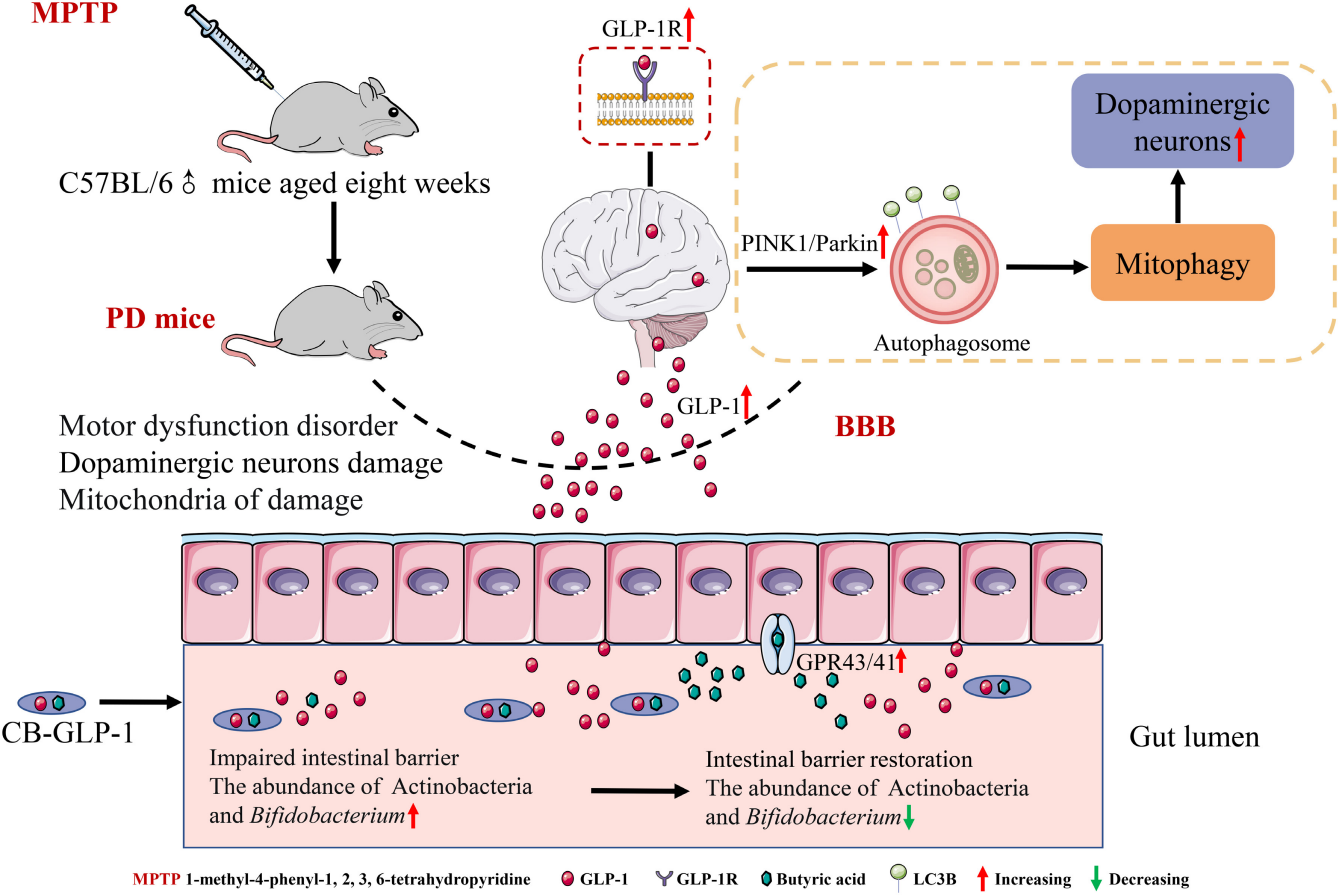
Schematic illustration of the potential mechanism by which *C. butyricum*‐GLP‐1 ameliorates PD. *C. butyricum*‐GLP‐1 could secrete GLP‐1, cross the blood–brain barrier into the brain, and act on the GLP‐1R to activate the mitophagy pathway to assert its neuroprotection against PD, and could enhance the intestinal mucosal barrier by rectifying the dysbiosis in the gastrointestinal tract. GLP‐1, glucagon‐like peptide 1; GLP‐1R, GLP‐1 receptor; PD, Parkinson's disease.

PD is a common neurodegenerative disorder, and there are currently no drugs available to stop or reverse its progression. In previous works, our team constructed two engineered strains MG1363‐GLP‐1 and *Escherichia coli* Nissle 1917‐GLP‐1 that could consistently express GLP‐1, and found that they significantly improved the behavioral characteristics of PD.^37^, ^38^ However, these two strains have the disadvantages of being resistant to drugs and weakly tolerant of the gastrointestinal tract, as well as being intolerant of storage once the product is formed. To overcome their limitations, in this study, an engineered bacterium was constructed using *C. butyricum* as a vector. Among other things, *C. butyricum* is a probiotic that promotes GLP‐1 levels in the colon and has a protective effect on the central nervous system. In general, we have constructed *C. butyricum*‐GLP‐1 engineering bacteria with great clinical application potential. First, it can not only continuously express GLP‐1, but also requires only oral administration and is relatively inexpensive, which can reduce or alleviate the pain or burden of patients. Simultaneously, because our engineered bacteria contain spores, they are more resistant to storage after forming products, which will help the subsequent industrialization of *C. butyricum*‐GLP‐1 (Figure 9).

As mentioned above, an engineered strain of *C. butyricum*‐pMTL007‐GLP‐1 was constructed. The result of microbial characteristics of *C. butyricum*‐GLP‐1 demonstrated that the construction of *C. butyricum*‐GLP‐1 engineered bacteria could secrete GLP‐1 well, with excellent ability of resisting acids, bile salts, and the potential to be taken orally (Figure 1).

Based on the good results of the previous experiments, we constructed the PD model mice using MPTP to investigate the impact and mechanism of *C. butyricum*‐GLP‐1 in PD. MPTP is one of the most commonly used neurotoxins in PD animal models, and MPTP is a lipophilic molecule. This allows it to easily cross the blood–brain barrier.^39^ Second, its use is not technically challenging, and finally, MPTP produces reliable and reproducible damage to the nigrostriatal dopaminergic pathway following systemic administration, so we chose MPTP to induce PD in the mouse model.^40^ Our results revealed that the M group had particularly longer descent time, lower suspension score, and significantly shorter total motor distance and distance in the central region. This was consistent with previous findings.^30^, ^41^ In contrast, *C. butyricum*‐GLP‐1 reversed these alterations, which demonstrated that *C. butyricum*‐GLP‐1 could alleviate MPTP‐induced motor dysfunction in PD (Figure 2).

TH, α‐syn, and DAT are crucial proteins in the development of PD. Our works discovered that TH‐positive cells were decreased in the SN and CS, and the positive cells of α‐syn were highly increased in the M group; similar results were obtained in Western blotting. In addition, we found a significant reduction in the DAT, which was reported in previous work.^42^ TH, a rate‐limiting enzyme in the synthesis of dopamine, is a protein marker of DAergic neurons.^43^ The main pathological feature of PD is the existence of Lewy bodies rich in α‐syn.^44^ Besides, DAT is an important sign of presynaptic DAergic endings, while the striatum's DAergic neuron endings synapses reflect the major pathological changes of PD in a concentrated manner.^45^ In contrast, *C. butyricum*‐GLP‐1 administration increased TH and DAT levels and decreased α‐syn aggregation, suggesting that *C. butyricum*‐GLP‐1 could prevent MPTP‐induced neuropathologic changes in PD mice (Figure 3).

Next, we explored the correlation between *C. butyricum*‐GLP‐1 improvement of motor dysfunction and neuropathological changes in PD with GLP‐1 expression. The results showed that GLP‐1 concentration in the SN was appreciably decreased in the M group. Furthermore, we detected GLP‐1R expression. Our study found that MPTP reduced the expression of GLP‐1R, which corresponded to prior studies.^46^ While treatment with *C. butyricum*‐GLP‐1 remarkedly restored GLP‐1R level, these results proposed that *C. butyricum*‐GLP‐1 may up‐regulate the GLP‐1 and GLP‐1R levels (Figure 4).

Next, we intended to explore the underlying mechanism through which *C. butyricum*‐GLP‐1 reduces motor dysfunction in PD. Mitophagy can eliminate damaged or extra mitochondria, allowing cells to function normally.^47^ Increasing work supports that enhancing PINK1/Parkin‐mediated mitophagy may be a therapeutic approach to PD. In addition, Lin et al. showed that liraglutide ameliorates PD by promoting mitophagy flux.^48^ This finding was supported by our results, which proved that the expression of Beclin‐1, PINK1, Parkin, Atg7, LC3B II, and LAMP‐1 were significantly inhibited, while p62 was substantially raised after MPTP treatment, with *C. butyricum*‐GLP‐1 administration greatly reversed the effect of MPTP. Related studies found that when mitochondria are damaged, Beclin‐l, a vital gene for autophagy, plays a major role in modulating autophagy, recruiting related proteins and key factors in inducing autophagy.^49^ Among them, PINK1, a mitochondrial kinase responsible for activating Parkin and transporting it from the cytoplasm to damaged mitochondria,^50^ which leads to ubiquitination and subsequently a cytoplasmic form of LC3 (LC3B I) binding to phosphatidylethanolamine via the activating enzymes ATG7 to form LC3B II,^51^ which degrades damaged mitochondria by recognizing the gap junction protein p62 and thus specifically linking to substrates such as damaged mitochondrial structural proteins by binding to lysosomes (LAMP1 is considered a lysosomal marker protein).^52^ In addition, the results were further supported by IF. Furthermore, we examined mitochondrial changes by TEM to support the above conclusion. The result found that the mitochondria of the M group were severely disrupted, with almost no autophagosomes and autophagic lysosomes. However, *C. butyricum*‐GLP‐1 increased the autophagic lysosome which is formed by fusing autophagosomes with lysosomes to degrade broken mitochondria.^53^ We found autophagosomes only in the C group, but not in the treatment group, therefore indicating that autophagosomes might have a crucial effect on PD, which may give new insights and pave the way for future treatments of PD. We also found that autophagic lysosomes were monitored in the CBG group, and considering that autophagosomes are downstream products of autophagosomes, the production of autophagosomes in the MPTP model group may be co‐dominated with both exogenous GLP‐1 and *C. butyricum*, which deserves to be further investigated in the future (Figure 5).

Mitochondrion is an important site for redox in the animal organism.^54^ Mitochondria damage can lead to OS and consequently PD. Conversely, mitophagy removes damaged mitochondria to prevent OS and cell death.^55^ This in turn improves the symptoms of PD. In our study, MPTP immensely decreased the level of SOD and GSH‐Px, and raised MDA level, which was consistent with previous work.^56^ MDA, a byproduct of lipid peroxidation, has been well established as an OS marker. SOD and GSH‐Px are antioxidant enzymes that eliminate oxygen free radicals and restore cell damage caused by them. Nevertheless, *C. butyricum*‐GLP‐1 could enhance the activity of SOD and GSH‐Px, and suppress the activity of MDA to antagonism OS. Our results proved that *C. butyricum*‐GLP‐1 may reduce OS to alleviate the corresponding symptoms of MPTP‐induced mice (Figure 6).

Some studies have found a correlation between intestinal microbiota disorder and PD. In addition, Chen et al. discovered that *C. butyricum* could regulate gut microbiota.^57^ Therefore, we explored whether *C. butyricum*‐GLP‐1 could improve the intestinal microbiota disorder caused by PD. Our work found that the α‐diversity of the intestinal microbiota was reduced in the M group while *C. butyricum*‐GLP‐1 vastly enhanced the α‐diversity. Moreover, results indicated that *C. butyricum*‐GLP‐1 treatment decreased the abundance of the phylum Actinobacteria and the genus *Bifidobacterium*. Although *Bifidobacterium* is commonly considered to be beneficial bacteria which may ameliorate abdominal pain and bloating in PD patients,^58^ however, several studies found a positive correlation in Actinobacteria and *Bifidobacterium* with PD index of abnormal inflammation (percent neutrophils, monocytes cell count/percentage, white blood cell count),^59^ which was consistent with our results (Figure 7).

*C butyricum* is a probiotic that mainly produces butyrate to act on G protein‐coupled receptors GPR41/43 to play a beneficial role.^60^ Previous study had found that *C. butyricum* could improve the motor symptoms of PD by acting on GPR41/43 to promote GLP‐1 secretion.^18^ In addition, studies have reported that butyrate can prevent intestinal inflammation and activate NLRP3 inflammasomes by binding to GPR43 to promote epithelial integrity.^61^ We further explored the effect of *C. butyricum*‐GLP‐1 on butyrate receptors in PD mice. Results revealed that the expression of GPR41/43 in the colon of M group were vastly reduced, while *C. butyricum*‐GLP‐1 treatment reversed these changes, similar to findings reported previously.^18^ Therefore, we concluded that *C. butyricum*‐GLP‐1 could promote the expression of GPR41/43, which is assumed to have a role in the pathogenesis of PD. Intestine tight junction protein ZO‐1 and occludin are crucial component of intestinal barrier.^62^ Our results proved that MPTP substantially decreased ZO‐1 and occludin expression, which was consistent with Sarkar et al.^63^ while *C. butyricum*‐GLP‐1 recovered the decrease of intestinal tight‐junction‐associated proteins. In conclusion, *C. butyricum*‐GLP‐1 may improve the intestinal symptoms of PD by promoting GPR41/43 and restoring intestinal permeability (Figure 8).

## 5 CONCLUSION

Overall, our results indicated that *C. butyricum*‐GLP‐1 could secrete GLP‐1, cross the blood–brain barrier into the brain, and act on the GLP‐1R to activate the mitophagy pathway, thus exerting its neuroprotective effects against PD, and could enhance the intestinal mucosal barrier by rectifying the dysbiosis in the gastrointestinal tract, which contributed to the improvement of PD. This is the first study to combine the probiotic properties of *C. butyricum* and GLP‐1 to assess the effects on PD. It should be noted that in this work, we only investigated that *C. butyricum*‐GLP‐1 was able to treat Parkinsonism via potentiating mitophagy, but the exact molecular mechanism of mitophagy induction and the specific relationship between gut microbiota and PD need to be further investigated. Moreover, TH immunohistochemical staining on both sides may be more accurate and the content of butyric acid should be detected.

## AUTHOR CONTRIBUTIONS

**Yun Wang:** Conceptualization (equal); data curation (lead); formal analysis (lead); investigation (equal); methodology (equal); project administration (lead); writing – original draft (lead); writing – review and editing (lead). **Wenjie Chen:** Data curation (equal); investigation (equal); methodology (equal); writing – review and editing (equal). **Yi‐yang Han:** Data curation (equal); investigation (equal); methodology (equal). **Xuan Xu:** Data curation (equal); writing – review and editing (equal). **Ai‐xia Yang:** Investigation (equal); methodology (equal). **Jing Wei:** Methodology (equal); validation (equal). **Daojun Hong:** Data curation (equal); validation (equal). **Xin Fang:** Conceptualization (lead); project administration (lead); writing – original draft (equal); writing – review and editing (lead). **Tingtao Chen:** Conceptualization (lead); project administration (lead); writing – original draft (equal); writing – review and editing (lead).

## CONFLICT OF INTEREST STATEMENT

The authors declare no conflicts of interest.

### PEER REVIEW

The peer review history for this article is available at https://www.webofscience.com/api/gateway/wos/peer-review/10.1002/btm2.10505.

## Supporting information


**Table S1.** Antibodies used in this study.

## Data Availability

Data will be made available on request.
