# Supplementary material for: Neuroprotective effect of engineered Clostridium butyricum‐pMTL007‐GLP‐1 on Parkinson's disease mice models via promoting mitophagy
Source: Bioeng Transl Med. 2023 Mar 17;8(3):e10505. doi: 10.1002/btm2.10505 (PMC10189449; doi:10.1002/btm2.10505)
Supplement: Supplementary file 1 — Table S1. Antibodies used in this study. [file BTM2-8-e10505-s001.pdf]

Supplementary Table 1. *Antibodies used in this study.*

Antibodies	Source/Cat. No.	Host	Dilution
Tyrosine hydroxylase<br>(TH)	Proteintech (25859-1-AP)	Rabbit	1 : 1,000 (WB)<br>1 : 1, 000 (IHC)
α-synuclein<br>(α-syn)	Proteintech (10842-1-AP)	Rabbit	1 : 1,000 (WB)<br>1 : 200 (IF)
Glucagon-like peptide 1<br>receptor<br>(GLP-1R)	Proteintech (26196-1-AP)	Rabbit	1 : 1,000 (WB)<br>1 : 500 (IHC)
Fatty acid receptor 3/GPR41<br>(GPR41)	Proteintech (66811-1-Ig)	Mouse	1 : 1,000 (WB)<br>1 : 200 (IHC)
GPR43	Thermo (PA5-111780)	Rabbit	1 : 200 (WB)<br>1 : 200 (IHC)
Zonula occludens-1<br>(ZO-1)	Proteintech (21773-1-AP)	Rabbit	1 : 500 (WB)
Occludin	Proteintech (13409-1-AP)	Rabbit	1 : 1, 000 (WB)
β-actin	CST (4970S)	Mouse	1 : 1, 000 (WB)
Dopamine transporter<br>DAT	Proteintech (22524-1-AP)	Rabbit	1 : 1, 000 (WB)
Parkin RBR E3<br>ubiquitin-protein ligase<br>(Parkin)	Proteintech (14060-1-AP)	Rabbit	1 : 1, 000 (WB)
LC3B	CST (43566)	Rabbit	1 : 1, 000 (WB)<br>1 : 6,400 (IF)
p62	CST (5114)	Rabbit	1 : 1, 000 (WB)
PTEN-induced kinase 1<br>(PINK1)	NOVUS (BC100-494)	Rabbit	1 : 1, 000 (WB)
Beclin-l	CST (3495S)	Rabbit	1 : 1, 000 (WB)
LAMP1	Abcam (ab208943)	Rabbit	1 : 1, 000 (WB)
Atg7	Abcam (ab133528)	Rabbit	1 : 1, 000 (WB)
